# In Loving Memory of Professor Kusum Jackson Nathoo 2 July 1943 – 23 June 2025

**DOI:** 10.4314/ahs.v26i1.17

**Published:** 2026-03

**Authors:** Patience Kuona

**Affiliations:** On behalf of Department of Child Adolescent and Women's Health, Faculty of Medicine and Health Sciences, University of Zimbabwe

**Figure:**
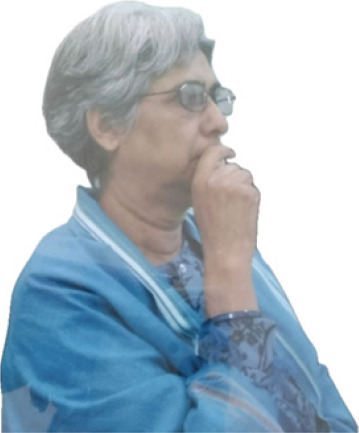


A dedicated clinician, educator, and researcher who served the University of Zimbabwe with unwavering commitment for over 46 years, having joined the Department of Paediatrics on 1st of January 1978 as a Lecturer. She was promoted to Senior Lecturer in 1987, Associate Professor in the same year, and attained the rank of Full Professor in 2003, serving as Professor of Paediatrics for 21 years.

Her academic qualifications included Bachelor of Medicine and Surgery (MBChB), Diploma in Child Health (DCH), Member of the Royal College of Physicians (MRCP), and a Master of Science in Clinical Epidemiology, reflecting her deep commitment to both clinical excellence and research. She served as a consultant paediatrician treating sick children at the then Harare Central Hospital now Sally Mugabe Children's Hospital for over 40 years. Her research work was characterized by a strong commitment to improving maternal and child health outcomes in resource-limited settings, with a particular emphasis on infectious diseases like HIV and vaccine-preventable illnesses, and the public health strategies required to address these challenges.

She served faithfully to the very end, giving of herself fully. A true heroine of Paediatric Medicine, she mentored many and built a lasting legacy of excellence, compassion, and selflessness. She has now rested, having run her race with honour. As we continue to serve in our time, we are reminded that our moment too shall come.

May her soul rest in eternal peace.

